# Prognostic importance of glycemic variability on left ventricular reverse remodeling after the first episode of ST-segment elevation myocardial infarction

**DOI:** 10.1186/s12933-023-01931-3

**Published:** 2023-08-04

**Authors:** Yohei Hanajima, Noriaki Iwahashi, Jin Kirigaya, Mutsuo Horii, Yugo Minamimoto, Masaomi Gohbara, Takeru Abe, Kozo Okada, Yasushi Matsuzawa, Masami Kosuge, Toshiaki Ebina, Kiyoshi Hibi

**Affiliations:** 1 Division of Cardiology, Yokohama City University Medical Center, 4-57 Urafune-cho, Minami-ku, Yokohama, 232-0024 Japan; 2 Department of Cardiology, Yokohama City University Graduate School of Medicine, Yokohama, Japan; 3 Department of Quality and Safety in Healthcare, Yokohama City University Medical Center, Yokohama, Japan

**Keywords:** Acute myocardial infarction, Continuous glucose monitoring system, Glucose, Glycemic variability, Remodeling

## Abstract

**Background:**

This study aimed to investigate the effect of glycemic variability (GV), determined using a continuous glucose monitoring system (CGMS), on left ventricular reverse remodeling (LVRR) after ST-segment elevation myocardial infarction (STEMI).

**Methods:**

A total of 201 consecutive patients with STEMI who underwent reperfusion therapy within 12 h of onset were enrolled. GV was measured using a CGMS and determined as the mean amplitude of glycemic excursion (MAGE). Left ventricular volumetric parameters were measured using cardiac magnetic resonance imaging (CMRI). LVRR was defined as an absolute decrease in the LV end-systolic volume index of > 10% from 1 week to 7 months after admission. Associations were also examined between GV and LVRR and between LVRR and the incidence of major adverse cardiovascular events (MACE; cardiovascular death, acute coronary syndrome recurrence, non-fatal stroke, and heart failure hospitalization).

**Results:**

The prevalence of LVRR was 28% (n = 57). The MAGE was independent predictor of LVRR (odds ratio [OR] 0.98, p = 0.002). Twenty patients experienced MACE during the follow-up period (median, 65 months). The incidence of MACE was lower in patients with LVRR than in those without (2% vs. 13%, p = 0.016).

**Conclusion:**

Low GV, determined using a CGMS, was significantly associated with LVRR, which might lead to a good prognosis. Further studies are needed to validate the importance of GV in LVRR in patients with STEMI.

## Background

Reperfusion therapy is an established treatment for ST-segment elevation myocardial infarction (STEMI). However, left ventricular adverse remodeling (LVAR) after STEMI still occurs in a significant proportion of patients and is not necessarily related to infarct size (IS). LVAR is associated with the development of heart failure and a poor prognosis [1, 2]. In contrast, left ventricular reverse remodeling (LVRR) after STEMI is associated with a good prognosis [3]. However, the precise mechanism underlying this remodeling remains unclear.

We previously reported an association between high glycemic variability (GV), determined using a continuous glucose monitoring system (CGMS), and LVAR after the first episode of STEMI [4]. Moreover, high GV is associated with a poor prognosis in patients with acute coronary syndrome (ACS) [5]. Therefore, we believe that GV plays an important role in both LVAR and LVRR.

However, there have been no studies on the association between LVRR and GV, and the role of GV in relation to changes in left ventricular (LV) structure and function has not yet been fully resolved. Therefore, this study evaluated the effect of GV on LVRR, as assessed using cardiac magnetic resonance imaging (CMRI), in patients with a first episode of STEMI.

## Methods

### Study population

**Fig. 1:**
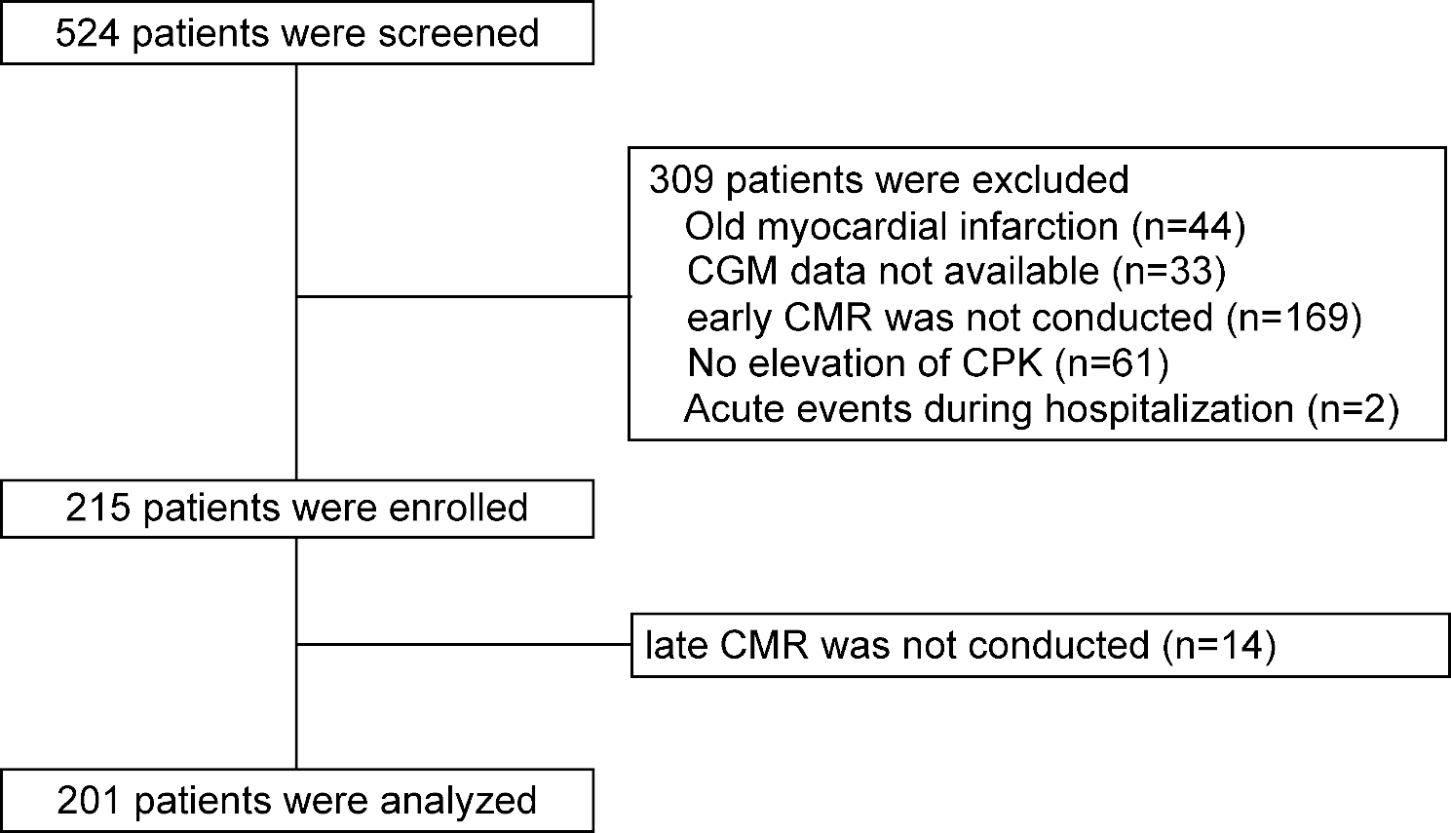
Study flow chart. The analysis included 201 patients

This study was conducted at the Yokohama City University Medical Center between April 2012 and March 2020. Figure 1 shows the patient enrollment flowchart. We screened 524 consecutive patients with STEMI who were successfully treated with percutaneous coronary intervention (PCI) within 12 h of symptom onset. They were fitted with a CGMS during hospitalization and underwent CMRI at 1 week and 7 months after STEMI. Patients fulfilling any of the following criteria were excluded: previous myocardial infarction (n = 44); maximum serum creatinine phosphokinase (CPK) levels were less than twice the upper limit of normal (n = 61); early CMRI was not conducted (n = 169); unavailable CGMS data (n = 33); acute adverse events, such as early stent thrombosis during hospitalization (n = 2); or late CMRI was not conducted (n = 14). The following criteria were used to define ST-segment elevation: new ST elevation at the J point in at least two contiguous leads of 2 mm in men or 1.5 mm in women in leads V2–3, or of 1 mm in other leads, or both. The new left bundle branch block was considered equivalent to STEMI. To ensure that LV remodeling was influenced by acute myocardial injury, we excluded patients with previous myocardial infarction. In total, 201 patients met the eligibility criteria and were enrolled in this study (Fig. 1).

All patients had a final thrombolysis in myocardial infarction (TIMI) flow grade of ≥ 2. The study protocol was approved by the Yokohama City University Medical Center Institutional Review Board, and written informed consent was obtained from all patients (UMIN-CTR ID: UMIN000010620).

### Biochemical markers

Peripheral blood samples, including blood glucose, CPK, creatine kinase MB (CK-MB), high-sensitivity C-reactive protein (hs-CRP), and brain natriuretic peptide (BNP) levels, were evaluated on admission, daily until discharge, and 1 month after the onset of STEMI. The levels of low-density lipoprotein (LDL) cholesterol, high-density lipoprotein (HDL) cholesterol, triglycerides, and glycosylated hemoglobin A1c (HbA1c) were evaluated on admission.

### CGMS protocol

**Fig. 2:**
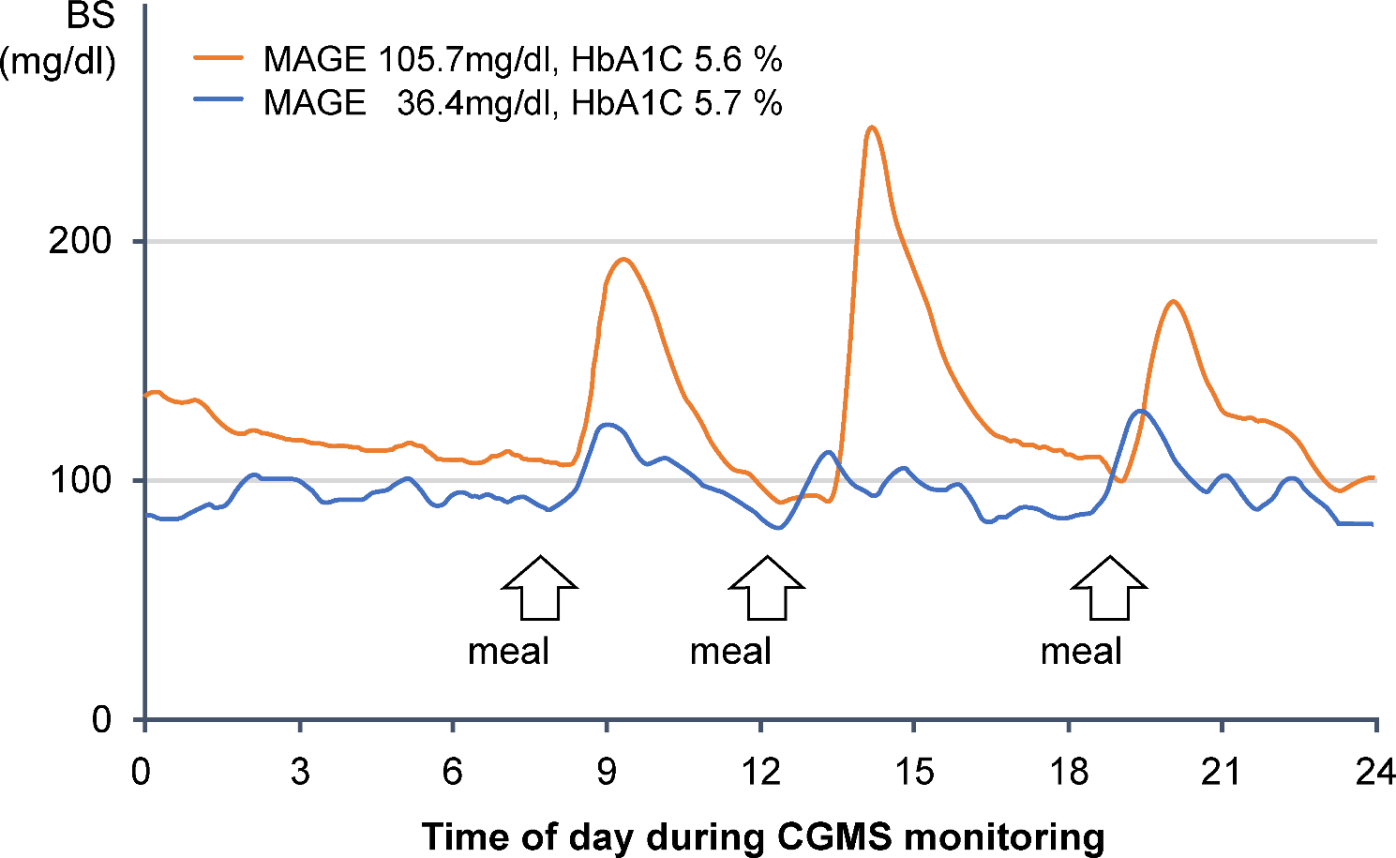
Representative cases of CGMS monitoring. Daily glucose fluctuation differed according to MAGE level

All patients were fitted with a CGMS (iPro2; Medtronic, Minneapolis, MN, USA) during hospitalization. The CGMS sensor was inserted into the subcutaneous abdominal fat tissue. During CGM, blood glucose levels were checked at least four times per day using a self-monitoring blood glucose device (Medisafe Mini, Terumo, Japan) to calibrate the CGMS data. The data obtained by the CGMS were recorded and analyzed offline. Five experienced observers interpreted the results. In addition to the mean amplitude of glycemic excursion (MAGE), the maximum, minimum, and average glucose levels were calculated. The MAGE was determined by calculating the arithmetic mean of the difference between consecutive peaks and the nadir if the difference was > 1 standard deviation of the mean glucose level [6]. MAGE analysis was performed at least 4 days after admission, considering stable dietary intake. Figure 2 shows representative examples of high- and low-MAGE cases. The patients had similar HbA1c levels, but daytime glucose fluctuations differed according to MAGE levels.

### 75 g oral glucose tolerance test (OGTT) protocol

After the patients’ conditions stabilized, almost all patients without diabetes mellitus (DM) underwent a 75 g oral glucose tolerance test (OGTT). After an overnight fast, venous blood samples for the measurement of plasma glucose levels were collected at baseline and 30, 60, and 120 min after an oral glucose load. DM, impaired glucose tolerance (IGT), and normal glucose tolerance (NGT) were classified according to the American Diabetes Association criteria [7].

### CMRI protocol

CMRI was performed twice using a 1.5-T CMRI system with an 8-element phased-array cardiac coil (MAGNETOM Avanto; Siemens Medical Solutions, Inc., Erlangen, Germany) at 1 week (early CMRI) and 7 months (late CMRI) after STEMI. Late gadolinium enhancement (LGE) images were acquired during the early phases.

After the scout imaging, cine true fast imaging with steady precession (True-FISP) sequences was obtained. Cine images were acquired in 6–8 short-axis views and long-axis views. The typical parameters were as follows: TR, 39.2 ms; TE, 1.94 ms; FA, 80°; slice thickness, 10 mm; matrix, 115 × 256; and FOV, 340 × 340 mm. Black blood T2-weighted CMRI images were acquired in the short-axis view with full left ventricular coverage. The typical parameters were as follows: TR, 2 R-R intervals; TE, 78 ms; FA, 180°; slice thickness, 10 mm; matrix, 148 × 256; and FOV, 340 × 340 mm. At 10–15 min after infusion of 0.1 mmol/kg gadolinium-diethylenetriamine pentaacetic acid (Gd-DTPA) (Magnevist, Bayer Schering Pharma, Berlin, Germany), LGE images were acquired using a phase-sensitive inversion recovery method in 6–8 short-axis views. The typical parameters were as follows: TR, 943.2 ms; TE, 1.33 ms; FA, 40°; slice thickness, 10 mm; matrix, 123 × 192; and FOV, 311.7 × 340 mm. All images were acquired during breath-holding at the end of expiration.

### CMRI analysis

All CMRI images were independently interpreted using Q-MASS MR 7.5 (Medis, Leiden, the Netherlands) by four experienced observers (Y.H., J.K., M.G., Y.M) who were blinded to the angiographic and clinical data.

After reviewing the cine images, left ventricular ejection fraction (LVEF), LV end-diastolic volume index (LVEDVI), and LV end-systolic volume index (LVESVI) were calculated by manually tracing the LV endocardial and epicardial borders on the short-axis images at end-diastole and end-systole.

Based on a recent study, LVRR was defined as an absolute decrease in LVESVI of > 10% on CMRI images obtained 1 week and 7 months after STEMI [3]. The IS and transmural extent of the infarct (TEI) were identified using LGE images in the early CMRI. The endocardial and epicardial borders were manually delineated on all the LGE short-axis LV images. A region of interest was then placed in the remote non-infarcted myocardium with uniform myocardial suppression. We used the full-width-at-half-maximum method to define the IS. LGE was defined as a signal intensity > 5 standard deviations above that of a remote non infarcted area in the same section. Microvascular obstruction (MVO) was defined as a dark area within the hyperenhanced area on LGE images and was considered to belong to the infarct area [8]. All measurements were calculated using the planimetric method and expressed in grams of myocardium. Values were normalized to the LV mass and presented as a percentage of LV mass.

TEI was also quantified by grading from 0 to 4: 0, no scar; 1, 1–25%; 2, 26–50%; 3, 51–75%; and 4, 76–100%. In this study, transmural infarction suggesting non-viable myocardium was defined as TEI grade 4 [9]. Myocardial edema (area at risk [AAR]) was defined on T2 weighted images as areas of high signal intensity exceeding the intensity of the remote non-infarcted myocardium by > 2 standard deviations. The mass of the edematous myocardium was calculated and presented as a percentage of the LV mass. The myocardial salvage index (MSI) was calculated as follows: AAR-IS/AAR [8].

### Long-term follow-up and definitions of major adverse cardiovascular events (MACE)

Patients were followed-up for a median period of 65 months (interquartile range [IQR], 43–90 months). During follow-up, we used composite MACE, defined as the occurrence of one of the following events: cardiac death, ACS recurrence, hospitalization for heart failure (HF), and stroke. All events were followed up with a hospital visit or telephone interview with an experienced cardiovascular physician who was blinded to the clinical details and outcomes.

### Statistical analysis

Continuous variables are expressed as median (25th to 75th percentiles) and categorical variables are reported as frequencies and percentages.

Differences between groups were tested using Student’s t-test for normally distributed variables and the Mann–Whitney test for variables with skewed distributions.

For categorical variables, Fisher’s exact test or the chi-square test was used, as appropriate. We used Cox proportional hazards models to evaluate associations between LVRR and the following variables: age, multivessel disease, initial TIMI flow grade > 1, final TIMI flow grade 3, ACE-I or ARB use, β blocker use, peak CPK level, CRP level at 1 month, MAGE, and CMRI parameters (IS, AAR, MSI, presence of MVO, and TEI = 4). We then established two different multivariable Cox proportional hazards models: model 1, with all variables included, and model 2, with variables with p values of < 0.2 in model 1 included. For sensitivity analysis, the association between reverse remodeling and time to MACE was assessed using Kaplan–Meier survival curves and the log-rank test. For all analyses, a two-tailed *p*-value < 0.05 was considered statistically significant. All analyses were performed using JMP, version 15.0.0 (SAS Institute Inc., Cary, NC, USA).

## Results

### Patient characteristics

A total of 201 patients were included in this analysis. Reverse remodeling (> 10% decrease in LVESVI at 7 months) occurred in 57 patients (28%). Patients were divided into two groups: patients with and without LVRR.

The baseline characteristics of the patients are shown in Table 1. The median age was 63 years (IQR 53–71 years), and 86% of the patients were males. The median time from symptom onset to reperfusion was 139 min (IQR 100–214 min). Clinical presentations such as Killip class, culprit artery, onset-to-reperfusion time, and initial and final TIMI flow grades were not different between the two groups. No significant differences were observed in medication use at follow-up (Table 1).

**Table 1:** Baseline Clinical Characteristics

	All	With LVRR (n = 57)	Without LVRR(n = 144)	p Value
Age, year	65 (53–71)	63 (51–71)	65 (55–72)	0.083
Male, n (%)	172 (86%)	48 (84%)	124 (86%)	0.730
Body mass index, kg/m²	24.5 (22.4–27.4)	25.6 (22.4–28.1)	24.1 (22.6–27.0)	0.861
Hypertension, n (%)	106 (53%)	29 (51%)	77 (54%)	0.740
Dyslipidemia, n (%)	162 (81%)	45 (79%)	117 (81%)	0.710
Killip class > 1, n (%)	14 (7%)	3 (5%)	11 (8%)	0.761
Infarcted artery				0.355
LAD culprit, n (%)	104 (52%)	34 (60%)	70 (49%)	
LCX culprit, n (%)	19 (9%)	5 (9%)	14 (10%)	
RCA culprit, n (%)	78 (39%)	18 (31%)	60 (41%)	
Multi vessel disease, n (%)	64 (32%)	17 (30%)	47 (32%)	0.700
Onset to reperfusion, min	139 (100–214)	144 (96–237)	136 (100–209)	0.432
Initial TIMI flow grade > 1, n (%)	45 (22%)	14 (25%)	31 (22%)	0.642
Final TIMI flow grade 3, n (%)	192 (96%)	56 (98%)	136 (94%)	0.450
**Laboratory data**				
LDL-C, mg/dl	131 (112-153.5)	137 (117–161)	127 (111–151)	0.133
HDL-C, mg/dl	42 (36–50)	41 (35–50)	42 (36–50)	0.632
Triglycerides, mg/dl	125 (76–220)	150 (98–227)	119 (71–202)	0.325
Peak level of CPK, IU/L	2397 (1329–4164)	2673 (1676–4482)	2286 (1206–4102)	0.476
Peak level of CPK-MB, IU/L	228 (129–404)	261 (181–422)	216 (118–400)	0.584
Peak level of CRP, mg/dl	6.14 (3.70–9.49)	5.21 (3.73–9.38)	6.59 (3.65–9.63)	0.694
CRP at 1 month, mg/dl	0.18 (0.08–0.42)	0.19 (0.09–0.43)	0.18 (0.07–0.43)	0.891
BNP at 1month, pg/ml	100 (49.6-199.1)	111.0 (64.8-193.3)	98.4 (46.5-202.2)	0.653
**Concomitant medication**				
Beta-blocker, n (%)	176 (88%)	52 (91%)	125 (86%)	0.384
ACE-I or ARB, n (%)	184 (91%)	50 (87%)	134 (93%)	0.220
Antidiabetic agents, n (%)	45 (22%)	13 (22%)	32 (22%)	0.929
Aspirin, n (%)	196 (97%)	56 (98%)	140 (97%)	0.675
Statin, n (%)	192 (96%)	57 (100%)	135 (93%)	0.054

LAD, left anterior descending artery; LCX, left circumflex artery; RCA, right coronary artery; CPK, creatine phosphokinase; CKMB, creatine phosphokinase-MB; BNP, brain natriuretic peptide; LDL, low-density lipoprotein; HDL, high-density lipoprotein; ACE-I, angiotensin-converting enzyme, ARB, angiotensin II receptor blocker

**Fig. 3:**
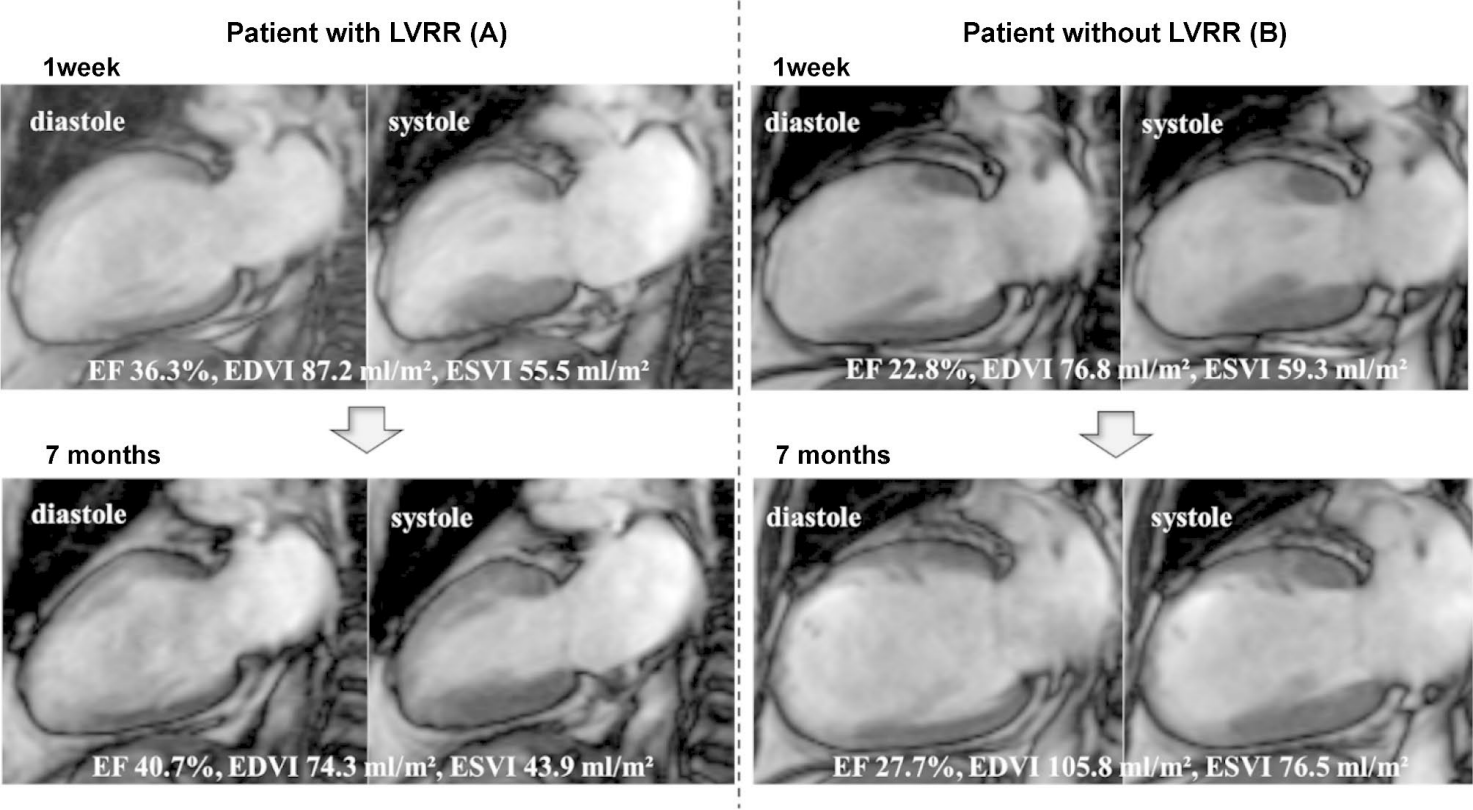
Representative cases of patients (**A**) with and (**B**) without LVRR. The two groups had similar peak levels of CPK (A: 4733 IU/L and B: 4798 IU/L) and HbA1c (A: 6.1% and B: 6.5%), but the MAGE differed (A: 26.5 mg/dl and B: 52.7 mg/dl)

The glucose metabolism and CGMS findings are shown in Table 2. No significant differences were seen in HbA1c levels and the profile of glucose metabolism disorders between the two groups. The MAGE was higher in patients with DM than in those without DM (64.1 ± 26.6 mg/dl vs. 42.9 ± 19.3 mg/dl, p < 0.001). The MAGE was lower in patients with LVRR than in those without LVRR (42.6 ± 26.3 vs. 54.6 ± 23.2 mg/dl, *p* < 0.001). The characteristics of the CMRI are listed in Table 2. In the early CMRI analysis, there were no significant differences in the IS, AAR, MSI, frequency of MVO presentation, or presence of transmural infarction between the two groups. The volumetric parameter changes in patients with and without LVRR from the early to late phases are shown in Table 3. In patients with LVRR, from early to late phase, LVEDVI and LVESVI decreased, and LVEF improved (p < 0.001 for all pairwise comparisons). In patients without LVRR, from early to late phase, LVEDVI and LVESVI increased (p < 0.001 for pairwise comparisons), and LVEF was maintained (p = 0.598 for pairwise comparison). Figure 3 shows the representative cases of patients with and without LVRR. They had anterior MI and similar CPK and HbA1c peak levels; however, the MAGE and course of structural change after STEMI were different.

**Table 2:** Characteristics of glucose metabolism and CMR parameter

	All	With LVRR (n = 57)	Without LVRR (n = 144)	p Value
Glucose metabolism, n(%)				0.401
DM	79 (39%)	20 (35%)	59 (41%)	
IGT	78 (39%)	21 (37%)	57 (40%)	
NGT	44 (22%)	16 (28%)	28 (19%)	
glucose on admission, mg/dl	156 (133–196)	152 (128–186)	158 (136–205)	0.263
HbA1c, %	5.9 (5.5–6.4)	5.8 (5.5–6.2)	5.9 (5.5–6.5)	0.518
**CGM parameter**				
MAGE, mg/dl	48 (32-63.9)	33.5 (24.1–54.9)	53.1 (38.6–68.3)	0.002
Max glucose level, mg/dl	170 (148–210)	151 (139–190)	179 (154–213)	0.044
Minimum glucose level, mg/dl	96 (83–104)	98 (82–104)	94 (85–105)	0.735
Incidence of hypoglycemia, n (%)	11 (5%)	3 (5%)	8 (6%)	1.000
Average glucose level, mg/dl	121 (112–136)	119 (110–127)	122 (112–138)	0.926
**early CMR**				
IS, % of LV mass	15 (9.75-22)	16 (11–24)	15 (9–22)	0.250
AAR, % of LV mass	27 (19–40)	29 (19–40)	26 (18–39)	0.214
MSI	0.44 (0.30–0.56)	0.41 (0.30–0.56)	0.44 (0.3–0.56)	0.717
Presence of MVO, n (%)	57 (42%)	22 (39%)	63 (44%)	0.505
Transmural infarction, n (%)	104 (52%)	25 (44%)	79 (55%)	0.160

DM, diabetes mellitus; IGT, impaired glucose tolerance, NGT, normal glucose tolerance; CGM, continuous glucose monitoring; MAGE, mean amplitude of glycemic excursion, CMR, cardiac magnetic resonance imaging; IS, infarct size; AAR, area at risk; MSI, myocardial salvage index; MVO, microvascular obstruction; LV, left ventricular; EF, ejection fraction; EDVI, end-diastolic volume index; ESVI, end-systolic volume index

**Table 3:** CMR volumetric parameter changes from 1 week to 7 months

	with LVRR (n = 57)				without LVRR (n = 144)		
	1 week	7 months	p value		1 week	7 months	p value
LVEF, %	42.7 (35.0-49.6)	51.1 (43.1–59.6)	< 0.001		46.5 (36.9–53.5)	47.2 (38.8–53.1)	0.598
LVEDVI, ml/m²	86.9 (72.9–103.0)	77.3 (65.9–89.9)	< 0.001		76.4 (62.9–87.2)	85.6 (72.2–98.1)	< 0.001
LVESVI, ml/m²	49.0 (39.2–60.0)	36.4 (29.2–45.7)	< 0.001		41.4 (30.7–52.6)	44.2 (33.7–56.9)	< 0.001

LV, left ventricular; EF, ejection fraction; EDVI, end-diastolic volume index; ESVI, end-systolic volume index

### Prediction of LV remodeling

Table 4 shows univariate and multivariate Cox proportional hazards models to characterize predictors of LVRR. Using multiple adjusted Cox proportional hazards analysis of model 2 to predict LVRR, MAGE could predict LVRR (odds ratio: OR 0.98, 95% CI 0.96 to 0.99, p = 0.002).

**Table 4:** Univariate and multivariate Cox proportional analysis to characterize predictors of the LVRR

Variables	Univariate Analysis			Multivariate Analysis (model1, forced)			Multivariate Analysis (model2, forced)		
	OR	95%CI	p Value	OR	95%CI	p Value	OR	95%CI	p Value
Age, per 1 year	0.98	0.96–1.01	0.166	0.97	0.94–1.01	0.100	0.98	0.95–1.00	0.102
Multi vessel disease, yes	0.88	0.45–1.71	0.698	0.87	0.40–1.93	0.740			
Initial TIMI flow grade > 1, yes	1.19	0.58–2.44	0.644	1.44	0.61–3.53	0.389			
Final TIMI flow grade 3, yes	3.39	0.40–26.95	0.266	7.55	0.66–62.89	0.108	5.58	0.63–49.26	0.122
ACE-I or ARB use, yes	0.53	0.19–1.48	0.226	0.38	0.12–2.09	0.340			
β blocker use, yes	1.58	0.56–4.46	0.387	2.05	0.43–6.14	0.472			
Peak level of CPK, per 100 IU/L	1.00	1.00–1.02	0.475	1.00	0.98–1.03	0.743			
CRP at 1month, per 0.1 mg/dl	0.95	0.88–1.01	0.118	0.93	0.86–1.01	0.103	0.93	0.86–1.01	0.069
HbA1C, per 0.1%	0.99	0.96–1.02	0.518	1.01	0.98 -1.05	0.416			
MAGE, per 1 mg/dl	0.98	0.96–0.99	0.002	0.97	0.95–0.99	0.002	0.97	0.96–0.99	0.002
IS, per 1%	1.02	0.99–1.05	0.250	1.00	0.84–1.19	0.984			
AAR, per 1%	1.01	0.99–1.03	0.214	1.03	0.93–1.13	0.577			
Myocardial salvage index, per 0.1	0.97	0.81–1.15	0.715	0.90	0.56–1.45	0.664			
Presence of MVO, yes	0.81	0.43–1.51	0.505	0.70	0.29-1.70	0.432			
Transmural infarction, yes	0.64	0.35–1.19	0.161	0.50	0.20–1.25	0.139	0.64	0.33–1.24	0.186

Abbreviations are described in Tables 1 and 2. CI, confidence interval; OR, odds ratio

### LV remodeling and MACE

**Fig. 4:**
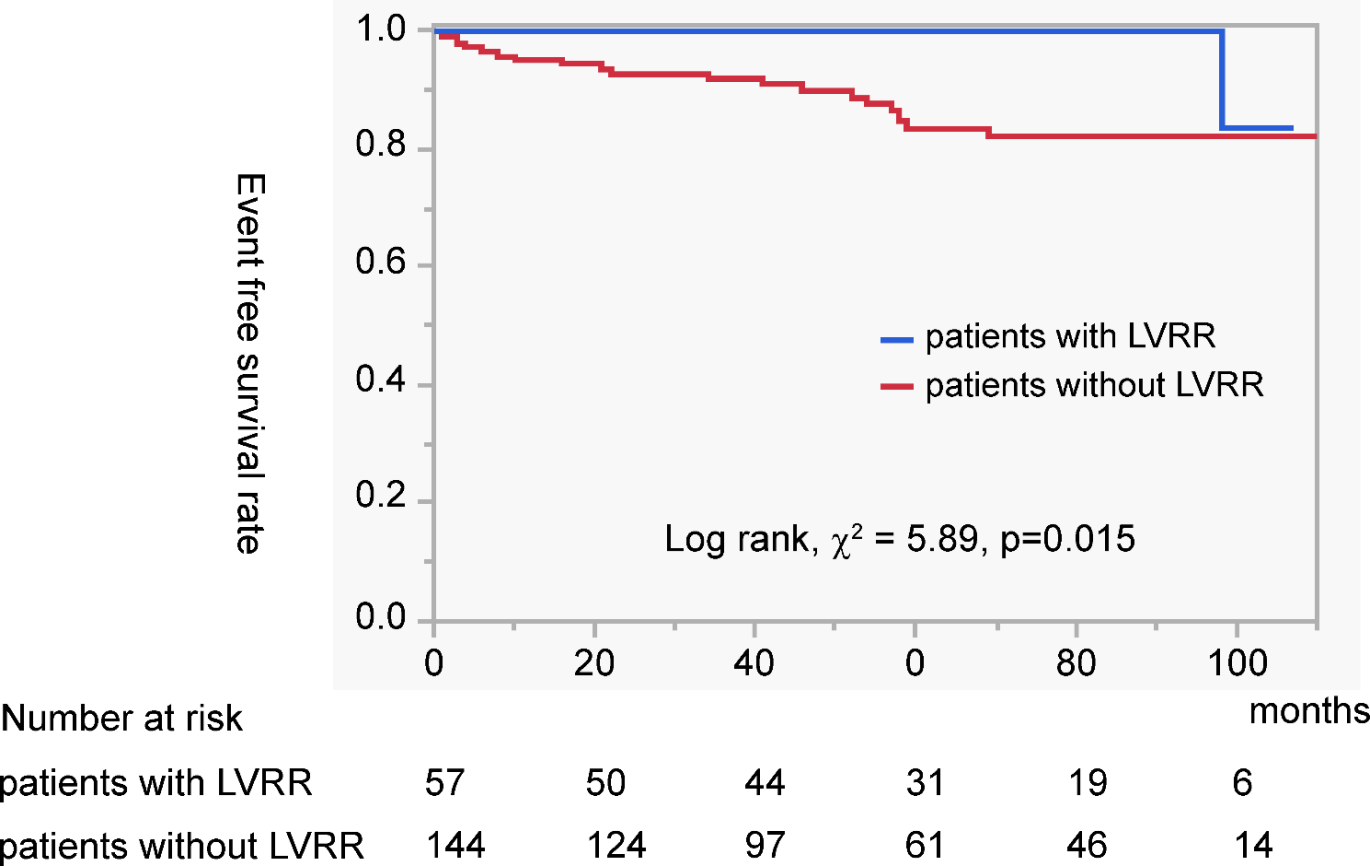
Kaplan–Meier curve analysis for MACE. Patients with LVRR had a significantly lower event free survival rate (p = 0.015)

During follow-up, 20 patients (10%) experienced MACE (Table 5). The incidence of MACE was significantly lower in patients with LVRR than in those without LVRR (2% vs. 13%, p = 0.016). The Kaplan–Meier curves for patients with LVRR are shown in Fig. 4. Patients with LVRR had significantly lower event-free survival rates (p = 0.015).

**Table 5:** Clinical events during follow-up

	All	With LVRR (n = 57)	Without LVRR(n = 144)	p Value
MACE	20 (10%)	1 (2%)	19 (13%)	0.016
Cardiovascular death, n (%)	1 (1%)	0 (0%)	1 (1%)	1.000
Recurrence of ACS, n (%)	9 (4%)	1 (2%)	10 (7%)	0.185
HF for hospitalization, n (%)	6 (3%)	0 (0%)	6 (4%)	0.186
Stroke, n (%)	6 (3%)	0 (0%)	6 (4%)	0.186
All cause death, n (%)	12 (6%)	4 (7%)	8 (6%)	0.744

MACE, major adverse cardiovascular event; ACS, acute coronary syndrome; HF, heart failure

## Discussion

In this study, we revealed that the MAGE evaluated using a CGMS could predict LVRR. Furthermore, this study showed that LVRR was associated with a good prognosis in patients with STEMI. Thus, GV may be an important factor in LV structural recovery and prognosis in patients with STEMI. To the best of our knowledge, this is the first study to reveal the role of GV, as evaluated using a CGMS, in LVRR after STEMI.

The following exceptional findings distinguish this study from existing literature. First, this study demonstrated the prognostic significance of GV in patients with mild glucose intolerance assessed using conventional parameters. In the present study, the average HbA1c level was relatively low (the median HbA1c level was 6.2%) because there was a limited number of patients with severe DM who underwent emergency PCI or were not treated for DM before the target hospitalization. Although the MAGE was higher in patients with DM than in those without DM in this study population, there were no significant differences in HbA1C levels and the distribution of the profile of glucose metabolism disorders between patients with LVRR and without LVRR. Thus, it should be emphasized that, in patients without severe DM, GV seems to be a stronger predictor of LVRR than HbA1c. Second, LVEF, LVEDVI, LVESVI, and IS were analyzed using CMRI. CMRI provides accurate and reproducible information on volumetric parameters and dynamic tissue changes, such as edema, MVO, and IS after STEMI [10]. Moreover, we examined the CMRI at 1 week and 7 months after the onset of STEMI. These serial examinations enabled us to precisely estimate LV remodeling. Third, to test the hypothesis that the myocardial healing process is influenced by GV, only patients with confirmed large acute MI were enrolled in this study. We excluded patients with STEMI with a maximum CPK level less than twice the upper limit of normal. Our results may be useful in the development of new evaluation strategies and possible treatments for patients with STEMI.

### Association between GV and LVRR

Recent studies have demonstrated that the prognostic significance of GV is superior to that of conventional impaired glucose tolerance parameters, such as HbA1c, fasting blood glucose, or admission glucose, in patients with ACS [5, 11]. The mechanisms underlying the effects of GV on clinical outcomes after STEMI are not fully understood. We previously reported the impact of GV on coronary plaque vulnerability, rapid progression of coronary plaque, and impaired left ventricular remodeling [4, 12–14]. These findings suggest that the degree of GV has a significant impact on changes in LV structure and function after STEMI.

Herein, we speculate on the mechanisms that may be responsible for the relationship between GV and LVRR. First, GV-induced oxidative stress may affect LV structural recovery after STEMI. GV was thought to be a specific trigger of oxidative stress, which contributes to the progression of inflammation and endothelial dysfunction, resulting in atherosclerosis [15–17]. Previously, we reported the clinical role of oxidative stress in patients with ACS [18]. In the context of myocardial remodeling following MI, dynamic tissue changes, such as myocardial fibrosis, hypertrophy, or apoptosis, occur to compensate for and respond to the loss of normal myocardium [19]. Oxidative stress plays a crucial role in these cellular changes [20]. Second, increased persistent activation of inflammatory pathways associated with GV may contribute to progressive adverse remodeling [1]. Although, multivariate analysis showed that the CRP level at 1 month was not significant predictor of LVRR in the present study, the trend was observed. In recent years, evidence has emerged that oxidative stress plays a crucial role in the development and perpetuation of inflammation, thus contributing to the pathophysiology of cardiovascular diseases [21]. Based on these findings, we speculate that low oxidative stress and subsequent inflammation suppressed by low GV may contribute to LVRR after STEMI. Unfortunately, we did not examine oxidative stress in the present study. In addition, we could not demonstrate a direct relationship between GV and inflammation. This may be due to the small sample size and the relatively mild degree of inflammation and oxidative stress in cases in which MRI and CGM were feasible. This issue should be addressed in future studies.

### Clinical implications

LVRR has been established as a good prognostic factor in patients with STEMI [3]. We believe that low GV might lead to LVRR after STEMI and contribute to a better prognosis. Various interventions, such as antidiabetic drug use, dietary interventions, and exercise training, have been reported [22].

In particular, a recent study showed that the use of sodium glucose co-transporter 2 inhibitors and a glucagon-like peptide-1 receptor agonist is associated with a reduction in GV [23]. Moreover, a recent trial showed that empagliflozin initiated shortly after AMI was associated with significant improvements in LVEF, LVEDV, and LVESV [24].

According to the results of the current study, GV may have a greater impact on prognosis than previously thought. Although HbA1c remains the gold standard for assessing glucose control, GV should also be considered as an additional parameter in the evaluation of glucose metabolism. We believe that GV could be a potential therapeutic target after STEMI.

### Study limitations

This study has several limitations. First, this was a single-center observational study. The external validity may be limited; therefore, caution should be exercised when applying the findings of this study. Second, there was selection bias in our study. Most cases were relatively mild, and the patients were able to wear the CGMS and start eating soon after STEMI. Moreover, we excluded patients who could not undergo CMRI because of kidney dysfunction. Chronic kidney disease (CKD) induces adverse cardiac remodeling, including left ventricular hypertrophy and cardiac fibrosis [25]. Nonetheless, this selection bias may reflect a relatively small number of MACE cases. Third, the use and choice of antidiabetic agents is at the discretion of doctors, and the prevalence of DM was 40%. Because the effects of antidiabetic agents were not considered in this study, an improvement in GV might have been observed in some patients using antidiabetic agents. Fourth, we did not investigate markers of oxidative stress. GV was thought to be associated with oxidative stress; however, the link between GV, evaluated using the MAGE, and oxidative stress remains unclear.

## Conclusion

In the first episode of STEMI, the MAGE is significantly associated with LVRR, which may lead to a good prognosis. Further studies are needed to validate the importance of GV in LVRR in patients with STEMI.

## Data Availability

Not applicable.

## List of abbreviations

AAR: Area at risk
ACE-I: Angiotensin converting enzyme inhibitor
ARB: Angiotensin receptor blocker
BNP: Brain natriuretic peptide
CGMS: Continuous glucose monitoring system
CKD: Chronic kidney disease
CMRI: Cardiac magnetic resonance imaging
CPK: Creatinine phosphokinase
DM: Diabetes mellitus
GV: Glycemic variability
HbA1c: Hemoglobin A1c
HDL: High density lipoprotein
HF: Heart failure hospitalization
hs-CRP: High-sensitivity C-reactive protein
IGT: Impaired glucose tolerance
IS: Infarct size
IS: Infarct size
LDL: Low-density lipoprotein
LGE: Late gadolinium enhancement
LVAR: Left ventricular adverse remodeling
LVEDVI: LV end-diastolic volume index
LVEF: Left ventricular ejection fraction
LVESVI: LV end-systolic volume index
LVRR: Left ventricular reverse remodeling
MACE: Major adverse cardiovascular events
MAGE: Mean amplitude of glycemic excursion
MSI: Myocardial salvage index
MVO: Microvascular obstruction
NGT: Normal glucose tolerance
OGTT: Oral glucose tolerance test
OR: odds ratio
PCI: Percutaneous coronary intervention
STEMI: ST-segment elevation myocardial infarction
TEI: Transmural extent of infarct
